# Development and Validation of an XGBoost-Based Machine Learning Model With Nomogram for Predicting Diabetic Peripheral Neuropathy Risk in Type 2 Diabetes Patients

**DOI:** 10.14740/jocmr6642

**Published:** 2026-07-31

**Authors:** Ning Yao, Ke Ke Zhang

**Affiliations:** a Department of Endocrinology, Mengcheng First People’s Hospital, Mengcheng, Anhui 233500, China

**Keywords:** Type 2 diabetes mellitus, Diabetic peripheral neuropathy, Machine learning, XGBoost, Random forest, Risk stratification model, Nomogram, SHAP

## Abstract

**Background:**

The study aimed to develop risk stratification models for diabetic peripheral neuropathy (DPN) in patients with type 2 diabetes mellitus (T2DM) using multiple machine learning algorithms, identify the optimal model, and visualize it through a nomogram, thereby providing a clinical decision-support tool for the early identification of high-risk individuals.

**Methods:**

A retrospective analysis was conducted on 180 inpatients diagnosed with T2DM at the Endocrinology Department of our hospital from January 2021 to September 2024, including 88 patients with DPN (48.9%) and 92 patients without DPN (51.1%). All enrolled participants were randomly divided into a training cohort (n = 126) and an internal validation cohort (n = 54) at a 7:3 stratified ratio. Collected clinical variables covered demographic profiles (age, gender, diabetes duration), anthropometric indicators (body mass index (BMI)), and multiple laboratory biomarkers. Five predictive algorithms were adopted for model construction, namely logistic regression (LR), random forest (RF), extreme gradient boosting (XGBoost), support vector machine (SVM), and decision tree (DT). Model predictive efficacy was comprehensively assessed using receiver operating characteristic (ROC) curves, calibration curves, decision curve analysis (DCA), and SHapley Additive exPlanations (SHAP) interpretability analysis. A visual nomogram was finally developed based on the best-performing model.

**Results:**

Multivariate regression analysis screened out six independent predictive factors for DPN occurrence, including diabetes duration, glycated hemoglobin (HbA1c), microalbuminuria (MAU), low-density lipoprotein cholesterol (LDL-C), neutrophil percentage (NEUT%), and BMI (all P < 0.05). Among all established models, the XGBoost algorithm yielded the best predictive outcomes in internal validation, with an area under the curve (AUC) of 0.903 (95% confidence interval (CI): 0.816–0.986), accuracy of 85.2%, sensitivity of 84.6%, and specificity of 85.7%. Its predictive efficacy was numerically superior to that of RF (AUC = 0.808), LR (AUC = 0.838), SVM (AUC = 0.884), and DT (AUC = 0.814). SHAP analysis further identified diabetes duration, HbA1c, and MAU as the most influential predictors for DPN risk. The nomogram established based on these core variables achieved a validation AUC of 0.85, with favorable calibration efficiency (Hosmer-Lemeshow P = 0.512) and positive net clinical benefit across a wide range of threshold probabilities.

**Conclusion:**

The XGBoost-based model shows favorable preliminary performance for cross-sectional DPN risk stratification in T2DM patients based on internal hold-out validation, outperforming traditional statistical approaches. Combined with SHAP interpretability and nomogram visualization, this model provides an exploratory clinical tool for early identification of potential high-risk individuals, requiring further external validation before clinical application.

## Introduction

Type 2 diabetes mellitus (T2DM) is a prevalent chronic metabolic disorder worldwide, imposing a growing socioeconomic burden on global public health systems. According to the 2021 report released by the International Diabetes Federation, the total number of diabetic patients globally has reached 537 million, and this figure is expected to exceed 783 million by 2045, with T2DM accounting for over 90% of all diabetes cases [1]. China bears the world’s largest diabetes population, with approximately 141 million affected individuals, and the prevalence of T2DM continues to rise rapidly [2]. Chronic complications induced by long-term hyperglycemia severely compromise patients’ quality of life and serve as major contributors to disability, premature mortality, and excessive medical resource consumption. As one of the most common and insidious microvascular complications of T2DM, diabetic peripheral neuropathy (DPN) is closely correlated with prolonged disease duration, suboptimal glycemic management, and complex metabolic disturbances [3].

DPN refers to peripheral nerve dysfunction occurring in diabetic patients after excluding other potential etiologies. Epidemiological investigations have demonstrated that 30% to 50% of T2DM patients may develop DPN during the disease course, and nearly 20% of patients present neurological lesions at the initial diagnosis of diabetes [4]. The clinical manifestations of DPN are heterogeneous and progressive with an insidious onset. Early-stage DPN is mainly characterized by distal sensory abnormalities such as numbness, burning sensation, and paresthesia. With disease progression, motor and autonomic nerve functions may be impaired, ultimately leading to foot ulceration, Charcot arthropathy, and lower extremity amputation. Clinical data indicate that more than 85% of diabetes-related amputations are secondary to diabetic foot ulcers, and DPN is recognized as the primary independent risk factor for ulcer formation [5]. Additionally, autonomic nerve involvement in DPN may reduce heart rate variability, induce orthostatic hypotension, and elevate the risk of sudden cardiac death, further worsening the long-term prognosis of diabetic patients [6].

The exact pathogenic mechanisms of DPN have not been fully elucidated, but existing studies have confirmed that its occurrence and progression result from the interaction of multiple molecular and metabolic pathways. Persistent hyperglycemia triggers excessive activation of the polyol pathway, leading to intracellular sorbitol accumulation, osmotic imbalance, and neuronal cytotoxic injury [7]. Meanwhile, the combination of advanced glycation end-products and their receptors activates the nuclear factor-κB inflammatory cascade, sustaining chronic neuroinflammation and damaging the structural integrity of myelin sheaths and axons [8]. Mitochondrial dysfunction and excessive reactive oxygen species accumulation further aggravate neuronal apoptosis via oxidative stress responses [9]. Moreover, impaired insulin signaling inhibits the secretion of neurotrophic factors such as nerve growth factor, weakening nerve repair capacity and rendering DPN an irreversible progressive disease once symptomatic lesions develop [10]. These complex multifactorial pathogenic mechanisms suggest that DPN cannot be explained by a single risk factor, but arises from the superposition of metabolic disorders, inflammatory activation, and impaired neuroprotective functions. This pathological feature supports the necessity of developing multi-variable integrated models for DPN risk evaluation.

Despite the deepening understanding of DPN pathogenesis, clinical early screening and precise risk stratification remain challenging. Conventional screening tools, including the Michigan Neuropathy Screening Instrument and Toronto Clinical Scoring System, rely heavily on manual physical examinations such as vibration perception, pain and temperature discrimination, and Achilles tendon reflex assessment. These methods are limited by subjective operational bias, poor repeatability, and insufficient sensitivity for detecting early subclinical DPN lesions [11]. Although nerve conduction velocity (NCV) testing is the gold standard for DPN diagnosis, its complicated operation and high detection cost restrict its large-scale popularization in routine clinical screening, especially in primary medical institutions [12]. Furthermore, most existing DPN prediction models are constructed based on traditional binary logistic regression (LR), with limited inclusion of predictive indicators. These models fail to fully integrate multidimensional metabolic and inflammatory biomarkers, resulting in restricted predictive accuracy and limited clinical applicability [13]. In current clinical practice, DPN is mostly diagnosed at advanced symptomatic stages, missing the optimal window for early intervention. Relevant studies have verified that standardized intensive glycemic control combined with comprehensive multi-risk intervention can reduce DPN incidence by nearly 50% [14]. Therefore, it is urgent to develop efficient, convenient, and accessible auxiliary tools for early DPN risk identification.

In recent years, machine learning (ML) algorithms have been widely applied in chronic disease risk prediction research. Owing to their unique advantages in capturing nonlinear relationships, processing high-dimensional clinical data, and conducting automatic feature screening, ML methods have become a novel and effective technical approach for medical risk stratification modeling [15]. Different from traditional statistical models that require strict data distribution assumptions, ML algorithms can adaptively identify complex interactions between clinical variables and mine latent disease correlation patterns in heterogeneous datasets [16]. Multiple mainstream ML algorithms, including random forest (RF), extreme gradient boosting (XGBoost), and support vector machine (SVM), have been successfully applied in predictive modeling for various chronic diseases with distinct advantages [17]. Ensemble learning models such as RF reduce overfitting risk through multi-tree integration, while XGBoost optimizes model performance via gradient boosting and regularization strategies, balancing predictive accuracy and model interpretability. At present, XGBoost has been widely used in risk prediction for diabetic nephropathy, retinopathy, and diabetic foot lesions [18]. Nevertheless, ML-based DPN risk stratification research is still relatively scarce. Existing related studies are limited by small sample sizes, incomplete variable screening, and lack of rigorous external validation, which greatly restrict their clinical application value [19].

Explainable artificial intelligence technology provides an effective solution to the inherent black-box defect of traditional ML models. As a mainstream interpretable tool, SHapley Additive exPlanations (SHAP) quantifies the independent contribution of each predictive feature to individual model outputs based on game theory principles, converting complex algorithmic decision logic into intuitive and quantifiable visual results. This technique effectively improves the transparency and clinical credibility of ML models, enabling clinicians to clearly understand the importance and action direction of different risk factors, and providing data support for individualized clinical intervention [20]. SHAP multi-dimensional visualization tools have been extensively adopted in cardiovascular disease, critical illness, and oncology prognostic research, while their systematic application in DPN risk assessment remains insufficient.

Currently, few published studies have integrated multi-algorithm ML comparison, SHAP interpretability analysis, and visual nomogram construction for DPN risk prediction. To fill this research gap, this study set three exploratory research objectives: (1) to systematically compare the preliminary predictive performance of five mainstream ML algorithms for DPN risk stratification; (2) to adopt SHAP analysis to clarify core predictive biomarkers and their nonlinear predictive characteristics; (3) to develop a clinically accessible nomogram based on optimal predictive features, so as to balance model predictive efficacy and clinical practicability and provide preliminary auxiliary reference for early DPN high-risk population screening.

## Materials and Methods

### Study population

This cross-sectional study enrolled patients with T2DM who were hospitalized in the Department of Endocrinology at our institution between January 2021 and September 2024. Inclusion criteria were as follows: (1) age ≥ 18 years; and (2) a confirmed diagnosis of T2DM according to the 2019 American Diabetes Association diagnostic criteria. Exclusion criteria included: (1) type 1 diabetes mellitus or other specific types of diabetes; (2) acute diabetic complications, such as diabetic ketoacidosis or hyperosmolar hyperglycemic state; (3) concurrent conditions potentially affecting peripheral nerve function, including vitamin B12 deficiency, thyroid dysfunction, chronic alcoholism, autoimmune diseases, or malignancy; (4) severe hepatic or renal insufficiency; and (5) incomplete clinical data. This study was approved by the institutional review board of the First People’s Hospital of Mengcheng County, which waived the requirement for informed consent due to the retrospective nature of the initial analysis. The study followed the tenets of the Declaration of Helsinki.

### Clinical data collection

Patient data were extracted from the electronic medical record system. Demographic characteristics collected included age, sex, and duration of diabetes. Anthropometric measurements comprised body mass index (BMI), calculated as weight divided by the square of height (kg/m^2^). Laboratory parameters included fasting plasma glucose (FPG), glycated hemoglobin (HbA1c), a comprehensive lipid profile (total cholesterol (TC), triglycerides (TG), high-density lipoprotein cholesterol (HDL-C), and low-density lipoprotein cholesterol (LDL-C)), hepatic and renal function indices, microalbuminuria (MAU), hemoglobin (HGB), platelet count (PLT), and neutrophil percentage (NEUT%).

### Diagnostic criteria for DPN

The diagnosis of DPN was established uniformly by two attending endocrinologists with over 5 years of specialized clinical experience, and disputed cases were adjudicated by a senior chief endocrinologist to ensure diagnostic consistency. The unified diagnostic criteria were as follows: (1) a confirmed history of diabetes mellitus; (2) onset of neuropathy either at the time of or subsequent to the diabetes diagnosis; (3) presence of clinical symptoms of neuropathy such as pain, numbness, or paresthesias accompanied by at least one abnormal finding among five standardized neurological tests (ankle reflex, vibration perception, pressure sensation, temperature discrimination, and pinprick pain sensation); in the absence of clinical symptoms, abnormality in at least two of the five tests was required for diagnosis; and (4) exclusion of neuropathy attributable to other causes, including neurotoxic medications, vitamin B12 deficiency, cervical or lumbar spondylosis, cerebral infarction, chronic inflammatory demyelinating polyneuropathy, hereditary neuropathies, vasculitis, infections, and metabolic neurotoxicity secondary to renal insufficiency.

Moreover, the severity of DPN was graded based on the validated Toronto Clinical Scoring System (TCSS), which comprehensively evaluates clinical symptoms, sensory examination and tendon reflex status: mild DPN (TCSS score 3–5 points): only mild sensory abnormalities without motor dysfunction; moderate DPN (TCSS score 6–8 points): obvious sensory disturbance and partial tendon reflex loss; severe DPN (TCSS score 9–11 points): severe sensory impairment, complete tendon reflex loss, or combined with motor nerve dysfunction. In terms of diagnostic modalities, all severe DPN patients and partial moderate DPN patients underwent NCV testing (the diagnostic gold standard for DPN), while all mild DPN patients and the remaining moderate DPN patients were diagnosed strictly through standardized clinical symptoms and neurological physical examination with thorough exclusion of secondary peripheral neuropathy causes.

### Dataset partitioning and data preprocessing

**Figure 1:**
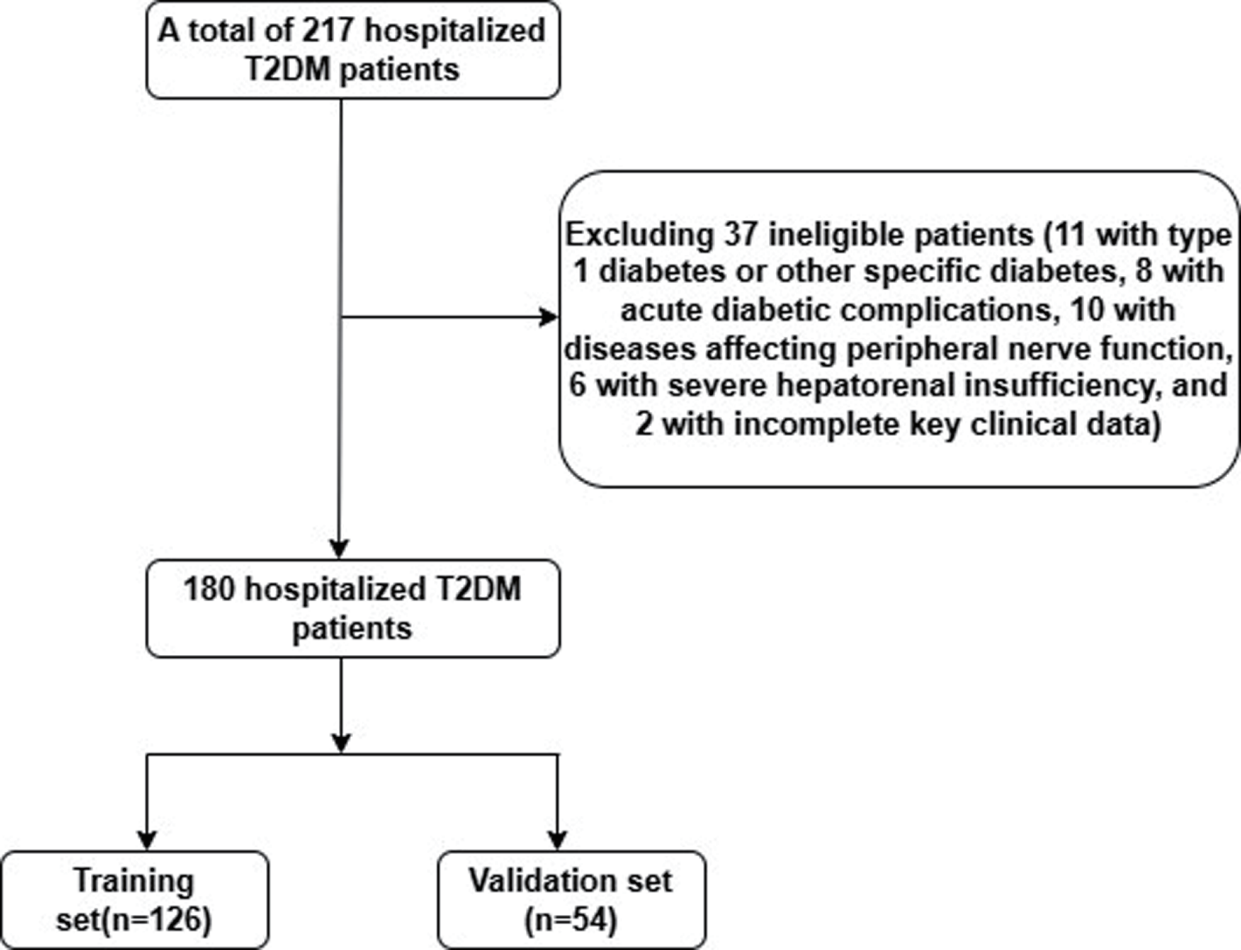
Study flow chart.

A detailed patient enrollment flowchart is presented in Figure 1. A total of 217 hospitalized T2DM patients were initially screened during the study period. After excluding 37 ineligible patients (11 with type 1 diabetes or other specific diabetes, eight with acute diabetic complications, 10 with diseases affecting peripheral nerve function, six with severe hepatorenal insufficiency, and two with incomplete key clinical data), a total of 180 patients were finally enrolled, including 88 DPN patients and 92 non-DPN patients. All enrolled patients were divided into a training set (n = 126,) and an internal hold-out validation set (n = 54) at a 7:3 stratified random ratio. The training set was used for model development and hyperparameter optimization, while the validation set was reserved for independent performance evaluation.

To ensure data quality and optimize model performance, a systematic data preprocessing strategy was implemented. For variables with a missing rate of less than 5%, multiple imputation was performed using the mice package in R, with five independent imputation iterations conducted to yield robust estimates. For variables with missing rates between 5% and 10%, complete case analysis was applied, supplemented by sensitivity analyses to assess the potential impact of missing data on study outcomes. In recognition of the differential sensitivity of ML algorithms to the scale of input features, a stratified preprocessing approach was adopted: all continuous variables were subjected to z-score standardization for the SVM and LR models to eliminate the influence of varying measurement units, whereas tree-based models including RF, XGBoost, and the decision tree (DT) were trained directly on raw continuous variables, capitalizing on the inherent scale-invariance of tree-based algorithms. Assessment of class imbalance revealed a near-equal distribution between the DPN group (88 cases, 48.9%) and the non-DPN group (92 cases, 51.1%), with an approximate 1:1 class ratio; therefore, data augmentation techniques such as the Synthetic Minority Over-sampling Technique were deemed unnecessary.

All data preprocessing and analytical procedures were implemented following a strict train-validation split workflow to eliminate data leakage. After completing stratified random partitioning of the training set and internal hold-out validation set, missing value imputation, variable z-score standardization, multi-strategy feature selection including univariable LR, least absolute shrinkage and selection operator (LASSO) regression and RF feature importance ranking, and model hyperparameter tuning were exclusively performed using only training set data. The internal hold-out validation set remained completely unseen during all preprocessing, feature screening, and model optimization processes and was only utilized for final model performance evaluation, which strictly avoids data leakage and ensures unbiased model validation results.

### Feature selection

A multi-strategy approach integrating three complementary methods was employed to identify clinically relevant predictors of DPN. First, univariable LR analysis was performed, and variables achieving statistical significance at P < 0.05 were retained as candidate features. Second, the mean decrease in Gini impurity was computed for each variable using RF, and features were ranked according to their importance scores. Third, LASSO regression was applied, with the optimal regularization parameter λ determined through 10-fold cross-validation, enabling the automatic elimination of redundant variables whose regression coefficients were shrunk to zero. This multi-dimensional feature selection framework integrates the complementary strengths of statistical significance testing, non-parametric importance ranking, and regularization-based variable selection, yielding a high-quality feature subset for subsequent ML model construction.

### ML model development and SHAP interpretability analysis

Five ML predictive models were developed in the training set using the selected feature variables. The LR model employed stepwise variable selection guided by the Akaike information criterion (AIC). The DT model utilized the Classification and Regression Tree (CART) algorithm, with optimal tree depth (range: 3–8) determined via 10-fold cross-validation. The RF model was based on a bootstrap aggregation strategy, with key hyperparameters including the number of trees (range: 100–500) and the maximum number of features optimized through grid search. The XGBoost model underwent Bayesian optimization to tune the learning rate (range: 0.01–0.3), tree depth (range: 3–8), subsampling rate (range: 0.6–1.0), and regularization parameters, with an early stopping strategy implemented to prevent overfitting. The SVM model employed a radial basis function (RBF) kernel, with the penalty parameter C (range: 0.1–100) and the kernel coefficient γ (range: 0.001–1) optimized via grid search. All models were evaluated using 10-fold cross-validation to ensure comparability and stability of results.

To enhance the interpretability of the optimal ML model, SHAP values were computed for each patient in both the training and validation sets, and three categories of visualizations were generated. The SHAP beeswarm plot illustrated the global ranking of feature importance and the directional influence of each feature on the predicted probability. The SHAP dependence plot elucidated the non-linear relationships between key features and predicted DPN risk. The SHAP waterfall plot provided individualized risk stratification explanations for single patients, quantifying the specific contribution of each feature to that patient’s risk assessment. This interpretability framework substantially enhances model transparency and provides a scientifically grounded basis for clinical decision-making.

### Nomogram construction and validation

A visual nomogram was constructed using the rms package in R, incorporating the six most important features identified by SHAP analysis in conjunction with the corresponding multivariable LR coefficients, to facilitate rapid bedside estimation of individual DPN risk. Model performance was evaluated across four dimensions: (1) discrimination the area under the ROC curve (AUC) with its 95% confidence interval (CI), along with accuracy, sensitivity, specificity, positive predictive value (PPV), negative predictive value (NPV), and F1 score; (2) calibration assessed using the Hosmer–Lemeshow goodness-of-fit test (P > 0.05 indicating adequate calibration) and a calibration curve generated with 1,000 bootstrap resamples; (3) clinical utility evaluated through decision curve analysis (DCA) to quantify net clinical benefit, supplemented by the net reclassification improvement (NRI) and integrated discrimination improvement (IDI) indices; and (4) model comparison conducted using the DeLong test to assess the statistical significance of AUC differences across models.

### Statistical software

All statistical analyses were performed using Python (version 3.10.0) and R (version 4.3.1). Key Python packages included scikit-learn (model construction), xgboost (XGBoost implementation), shap (SHAP analysis), optuna (Bayesian hyperparameter optimization), and matplotlib/seaborn (data visualization). Key R packages included rms (nomogram construction), pROC (ROC analysis), rmda (DCA), mice (multiple imputation), and ggplot2 (data visualization). All statistical tests were two-sided, and a P-value of less than 0.05 was considered to indicate statistical significance.

## Results

### Baseline characteristics of the study population

A total of 180 patients with T2DM were included in the final analysis, comprising 88 patients in the DPN group (48.9%) and 92 in the non-DPN group (51.1%). The training set included 126 patients (62 with DPN and 64 without DPN), and the validation set comprised 54 patients (26 with DPN and 28 without DPN). The median duration of diabetes in the DPN group was 9.5 years, significantly longer than the 5.2 years observed in the non-DPN group (P < 0.001). Compared with the non-DPN group, the DPN group exhibited significantly higher levels of HbA1c (9.2±1.8% vs. 7.8±1.4%, P < 0.001), MAU (89.5 vs. 28.6 mg/24 h, P < 0.001), and NEUT% (68.4±9.2% vs. 63.2±8.5%, P < 0.001), while HDL-C (1.08 ± 0.28 vs. 1.22 ± 0.30 mmol/L, P = 0.002) and HGB (128.5 ± 18.6 vs. 136.4 ± 17.2 g/L, P = 0.005) were significantly lower (Table 1).

**Table 1:** Comparison of Baseline Characteristics Between DPN and Non-DPN Groups

Variables	Non-DPN group (n = 92)	DPN group (n = 88)	P-value
Demographic characteristics			
Age (years), mean ± SD	56.8 ± 11.2	61.4 ± 10.6	0.006*
Male, n (%)	52 (56.5)	54 (61.4)	0.505
Duration of diabetes (years), mean (IQR)	5.2 (2.5–9.0)	9.5 (5.0–15.0)	< 0.001*
Clinical measurements			
BMI (kg/m^2^), mean ± SD	25.4 ± 3.2	26.8 ± 3.6	0.008*
Blood glucose metabolism			
FPG (mmol/L), mean ± SD	8.6 ± 2.8	9.8 ± 3.1	0.008*
HbA1c (%), mean ± SD	7.8 ± 1.4	9.2 ± 1.8	< 0.001*
Lipid profile			
TC (mmol/L), mean ± SD	4.52 ± 1.02	4.78 ± 1.18	0.124
TG (mmol/L), mean (IQR)	1.62 (1.12–2.38)	1.98 (1.28–2.85)	0.035*
HDL-C (mmol/L), mean ± SD	1.22 ± 0.30	1.08 ± 0.28	0.002*
LDL-C (mmol/L), mean ± SD	2.68 ± 0.82	3.05 ± 0.96	0.007*
Hepatic function			
ALT (U/L), mean (IQR)	22.0 (15.0–35.0)	24.5 (16.0–40.0)	0.326
AST (U/L), mean (IQR)	20.0 (14.0–28.0)	22.0 (16.0–32.0)	0.213
Renal function			
SCr (µmol/L), mean (IQR)	68.2 (56.4–82.6)	78.5 (62.0–98.4)	0.004*
BUN (mmol/L), mean ± SD	5.8 ± 1.6	6.5 ± 2.0	0.013*
eGFR (mL/min/1.73 m^2^), mean ± SD	88.4 ± 18.2	78.6 ± 20.5	0.001*
Urine parameters			
MAU (mg/24 h), mean (IQR)	28.6 (14.2–58.5)	89.5 (38.6–185.2)	< 0.001*
Blood routine			
HGB (g/L), mean ± SD	136.4 ± 17.2	128.5 ± 18.6	0.005*
PLT (× 10^9^/L), mean ± SD	218.5 ± 58.6	228.4 ± 65.2	0.299
NEUT% (%), mean ± SD	63.2 ± 8.5	68.4 ± 9.2	< 0.001*
Hypoglycemic medication, n (%)			
Single oral hypoglycemic drugs	34 (37.0)	21 (23.9)	0.062
Combined oral hypoglycemic drugs	29 (31.5)	24 (27.3)	0.564
Insulin monotherapy	16 (17.4)	23 (26.1)	0.168
Insulin + oral hypoglycemic drugs	13 (14.1)	20 (22.7)	0.139
DPN severity, n (%)			
Mild	-	42 (47.7)	-
Moderate	-	33 (37.5)	-
Severe	-	13 (14.8	-

*P < 0.05. Independent sample *t*-test or Mann–Whitney U test was used for continuous variables and χ^2^ test for categorical variables. ALT: alanine aminotransferase; AST: aspartate aminotransferase; BMI: body mass index; BUN: blood urea nitrogen; DPN: diabetic peripheral neuropathy; eGFR: estimated glomerular filtration rate; FPG: fasting plasma glucose; HbA1c: glycated hemoglobin; HDL-C: high-density lipoprotein cholesterol; HGB: hemoglobin; IQR: interquartile range; LDL-C: low-density lipoprotein cholesterol; LR: logistic regression; MAU: microalbuminuria; NEUT%: neutrophil percentage; PLT: platelet count; SCr: serum creatinine; SD: standard deviation; TC: total cholesterol; TG: triglycerides.

### Univariable LR and feature selection results

**Figure 2:**
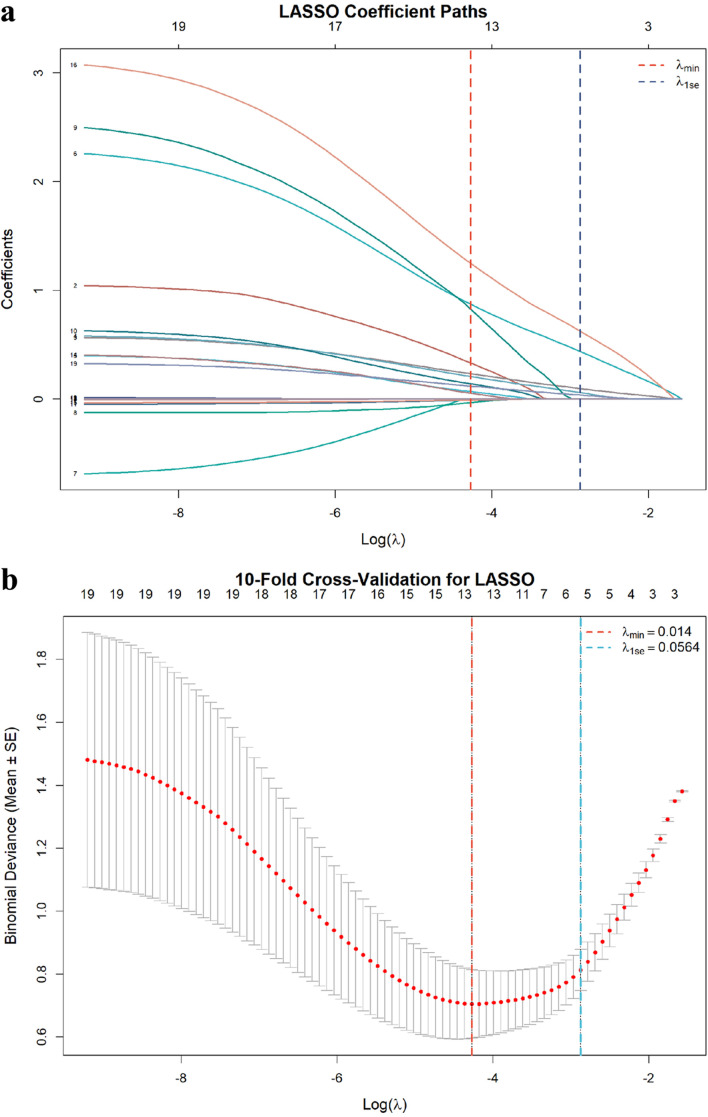
LASSO regression coefficient path diagram (a) and cross-validation error curve (b). The red dashed line indicates the optimal λ = 0.042, which corresponds to retaining eight non-zero coefficient features. LASSO: least absolute shrinkage and selection operator.

**Figure 3:**
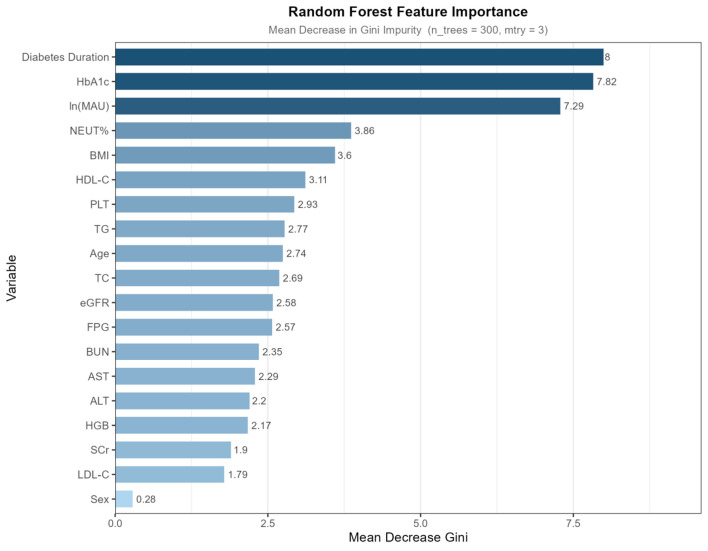
Random forest feature importance ranking chart (based on mean decrease Gini).

Univariable LR analysis (Table 2) identified 14 variables significantly associated with DPN occurrence (all P < 0.05): age, duration of diabetes, BMI, FPG, HbA1c, TG, HDL-C, LDL-C, serum creatinine (SCr), blood urea nitrogen (BUN), estimated glomerular filtration rate (eGFR), MAU, HGB, and NEUT%. Hypoglycemic medication types showed no significant correlation with DPN (all P > 0.05), as well as alanine aminotransferase (ALT), aspartate aminotransferase (AST), TC, and PLT (all P > 0.05). LASSO regression, with the optimal regularization parameter λ = 0.042 determined via 10-fold cross-validation, retained eight variables duration of diabetes, HbA1c, MAU, LDL-C, NEUT%, BMI, eGFR, and HGB while the coefficients of the remaining variables were shrunk to zero (Fig. 2). RF-based feature importance ranking (Fig. 3) identified the top eight features in descending order of importance as: duration of diabetes > HbA1c > MAU > NEUT% > LDL-C > eGFR > BMI > HGB. Synthesizing the results of all three feature selection methods, six core predictive variables were ultimately selected for model construction: duration of diabetes, HbA1c, MAU, LDL-C, NEUT%, and BMI.

**Table 2:** Univariate Logistic Regression Analysis of Risk Factors for DPN

Variables	β	OR	95% CI	P-value
Age (years)	0.042	1.043	1.012–1.075	0.007*
Gender (male vs. female)	0.182	1.200	0.614–2.344	0.592
Duration of diabetes (years)	0.148	1.160	1.095–1.228	< 0.001*
BMI (kg/m^2^)	0.128	1.137	1.036–1.247	0.007*
FPG (mmol/L)	0.138	1.148	1.042–1.264	0.005*
HbA1c (%)	0.524	1.689	1.386–2.057	< 0.001*
TC (mmol/L)	0.218	1.243	0.938–1.647	0.130
TG (mmol/L)	0.288	1.334	1.038–1.714	0.025*
HDL-C (mmol/L)	−1.648	0.192	0.082–0.451	< 0.001*
LDL-C (mmol/L)	0.442	1.556	1.164–2.080	0.003*
ALT (U/L)	0.008	1.008	0.993–1.024	0.282
AST (U/L)	0.012	1.012	0.993–1.032	0.226
SCr (µmol/L)	0.012	1.012	1.002–1.022	0.014*
BUN (mmol/L)	0.184	1.202	1.036–1.395	0.015*
eGFR (mL/min/1.73 m^2^)	−0.022	0.978	0.963–0.993	0.004*
MAU (mg/24 h)^a^	0.682	1.978	1.512–2.587	< 0.001*
HGB (g/L)	−0.018	0.982	0.968–0.997	0.018*
PLT (× 10^9^/L)	0.003	1.003	0.997–1.009	0.322
NEUT% (%)	0.068	1.070	1.033–1.109	< 0.001*

^a^MAU was included in regression analysis (ln-MAU) after natural logarithmic transformation. *P < 0.05 was included in multivariate analysis candidate set. ALT: alanine aminotransferase; AST: aspartate aminotransferase; BMI: body mass index; BUN: blood urea nitrogen; CI: confidence interval; DPN: diabetic peripheral neuropathy; eGFR: estimated glomerular filtration rate; FPG: fasting plasma glucose; HbA1c: glycated hemoglobin; HDL-C: high-density lipoprotein cholesterol; HGB: hemoglobin; IQR: interquartile range; LDL-C: low-density lipoprotein cholesterol; LR: logistic regression; MAU: microalbuminuria; NEUT%: neutrophil percentage; OR: odds ratio; PLT: platelet count; SCr: serum creatinine; SD: standard deviation; TC: total cholesterol; TG: triglycerides.

### Multivariable LR analysis

Multivariable binary LR analysis incorporating the six core predictive variables confirmed that duration of diabetes, HbA1c, MAU (natural log-transformed), LDL-C, NEUT%, and BMI were each independently associated with DPN (all P < 0.05) (Table 3). The variance inflation factor (VIF) for all variables was less than 3.0, indicating the absence of substantial multicollinearity. The Hosmer–Lemeshow goodness-of-fit test yielded χ^2^ = 7.486 (P = 0.486), confirming adequate model calibration.

**Table 3:** Multivariate Logistic Regression Analysis of Independent Risk Factors for DPN

Independent predictors	β	OR	95% CI	P-value	VIF
Duration of diabetes (years)	0.118	1.125	1.052–1.203	0.001	1.42
HbA1c (%)	0.406	1.501	1.196–1.883	< 0.001	1.68
ln-MAU (mg/24 h)	0.558	1.747	1.286–2.373	< 0.001	1.35
LDL-C (mmol/L)	0.382	1.465	1.062–2.021	0.020	1.28
NEUT% (%)	0.056	1.058	1.016–1.101	0.006	1.22
BMI (kg/m^2^)	0.102	1.107	1.012–1.212	0.026	1.18
Constant term	−16.428	-	-	< 0.001	-

BMI: body mass index; CI: confidence interval; DPN: diabetic peripheral neuropathy; HbA1c: glycated hemoglobin; LDL-C: low-density lipoprotein cholesterol; MAU: microalbuminuria; NEUT%: neutrophil percentage; OR: odds ratio.

### Hyperparameter optimization results

Following Bayesian optimization, the optimal hyperparameters for the XGBoost model were determined as follows: learning_rate = 0.06, max_depth = 4, n_estimators = 320, subsample = 0.82, colsample_bytree = 0.78, L1 regularization (alpha) = 0.12, and L2 regularization (lambda) = 1.58, min_child_weight = 3. The optimal hyperparameters for the RF model were: n_estimators = 300, max_depth = 6, min_samples_split = 5, and max_features = 'sqrt'. For the SVM model, the optimal parameters were: C = 12.6, γ = 0.042, and kernel = 'rbf'. For the DT model: max_depth = 5, min_samples_leaf = 8, and criterion = 'gini'. All models demonstrated stable performance across 10-fold cross-validation in the training set (Table 4).

**Table 4:** Performance of 10-Fold Cross-Validation for Five Machine Learning Models

Models	AUC (mean ± SD)	Accuracy (%)	Sensitivity (%)	Specificity (%)	F1 score
XGBoost	0.912 ± 0.048	85.7	84.2	87.2	0.858
Random forest	0.896 ± 0.052	83.3	82.6	84.0	0.836
Logistic regression	0.868 ± 0.058	80.2	79.5	81.0	0.802
SVM	0.845 ± 0.062	78.6	77.8	79.5	0.784
Decision tree	0.812 ± 0.072	76.2	75.4	77.0	0.760

AUC: area under the curve; SD: standard deviation; SVM: support vector machine.

### Comparative model performance in the validation set

The detailed performance metrics of all five models in the internal hold-out validation set (n = 54) are presented in Table 5. The XGBoost model achieved the highest preliminary overall performance for DPN risk stratification, with an AUC of 0.903 in the internal hold-out validation set, closely approximating the training set cross-validation AUC of 0.912; the marginal training-to-validation AUC difference of 0.009 indicated good internal generalization without evident overfitting, though these findings should be interpreted cautiously due to the limited validation sample size. DeLong test comparisons revealed that the AUC of XGBoost was significantly superior to those of LR (Z = 2.14, P = 0.033), SVM (Z = 2.68, P = 0.007), and DT (Z = 3.25, P = 0.001). Although no statistically significant difference in AUC was observed between XGBoost and RF (Z = 1.02, P = 0.307), XGBoost consistently outperformed RF across all other comprehensive performance metrics, including accuracy, sensitivity, specificity, and F1 score. It is worth noting that limited by the small sample size of the internal hold-out validation set (n = 54), the 95% CIs of model AUCs are relatively wide, and the current model performance ranking and statistical difference results may have certain instability, which should be interpreted cautiously. Although no statistically significant difference in AUC was observed between XGBoost and RF (Z = 1.02, P = 0.307), XGBoost consistently outperformed RF across all other comprehensive performance metrics, which needs further verification in large-sample cohorts.

**Table 5:** Performance Comparison of Five Machine Learning Models on the Validation Set

Models	AUC (95% CI)	Accuracy (%)	Sensitivity (%)	Specificity (%)	PPV (%)	NPV (%)	F1 score	TP	FP	TN	FN
XGBoost	0.903 (0.816–0.986)	85.2	84.6	85.7	84.6	85.7	0.846	22	4	24	4
Random forest	0.808 (0.713–0.895)	83.3	80.8	85.7	84.0	82.8	0.824	20	5	23	6
Logistic regression	0.838 (0.755–0.957)	79.6	80.8	78.6	77.8	81.5	0.793	19	6	22	7
SVM	0.884 (0.726–0.988)	77.8	76.9	78.6	76.9	78.6	0.769	21	5	23	5
Decision tree	0.814 (0.683–0.933)	74.1	73.1	75.0	73.1	75.0	0.731	18	7	21	8

AUC: area under the curve; CI: confidence interval; FN: false negatives; FP: false positives; NPV: negative predictive value; PPV: positive predictive value; SVM: support vector machine; TN: true negatives; TP: true positives.

### SHAP interpretability analysis of the XGBoost model

**Figure 4:**
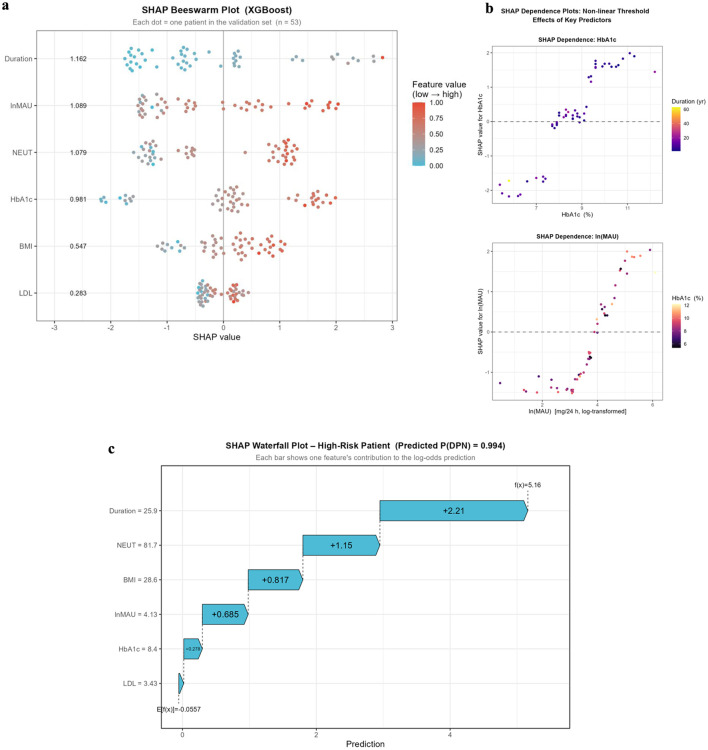
SHAP analysis results. (a) SHAP heat map displays the global importance ranking of each feature and the direction of their impact (red = high feature value, blue = low feature value, positive x-axis values = increased DPN risk); (b) SHAP dependence plots for HbA1c and MAU show non-linear effects and threshold phenomena; (c) SHAP waterfall plot for individualized risk stratifications in a single patient example (high-risk patient case). DPN: diabetic peripheral neuropathy; HbA1c: glycated hemoglobin; MAU: microalbuminuria; SHAP: SHapley Additive exPlanations.

The SHAP beeswarm plot (Fig. 4a) revealed the following feature importance ranking for the XGBoost model’s risk stratifications of DPN risk in the validation set: duration of diabetes > ln(MAU) > NEUT% > HbA1c > BMI > LDL-C. Color-coding further demonstrated that longer disease duration, elevated MAU, higher NEUT%, and increased HbA1c levels were each associated with a substantially greater predicted DPN risk.

The SHAP dependence plots (Fig. 4b) elucidated the non-linear relationship patterns of key features. The HbA1c dependence plot revealed a pronounced threshold effect: HbA1c values below 7% were associated with a protective effect, whereas values exceeding 8% corresponded to a sharp increase in DPN risk, with evidence of a synergistic interaction with disease duration. The ln(MAU) dependence plot exhibited an S-shaped curve, with SHAP values rising markedly when MAU exceeded 50 mg/24 h, indicating a distinct biological threshold for MAU.

The SHAP waterfall plot (Fig. 4c) illustrated the individualized risk stratification explanation for a representative high-risk patient. This patient had a diabetes duration of 25.9 years, which contributed the largest positive SHAP value (+2.21), followed by elevated NEUT% (+1.15) and overweight BMI (+0.817); MAU and poor HbA1c control contributed SHAP values of +0.685 and +0.278, respectively. The waterfall plot clearly visualized the cumulative feature contributions, yielding a final predicted DPN probability of 0.994, thereby providing a quantitative basis for the formulation of targeted individualized interventions.

### Net reclassification improvement and integrated discrimination improvement

Compared with the traditional LR model, the XGBoost model demonstrated significant improvements in reclassification and discrimination ability in the validation set, with an NRI of 0.248 (95% CI: 0.086–0.410, P = 0.003) and an IDI of 0.082 (95% CI: 0.026–0.138, P = 0.004), confirming the incremental predictive value of the ML approach over conventional statistical modeling.

### Nomogram construction and validation

**Figure 5:**
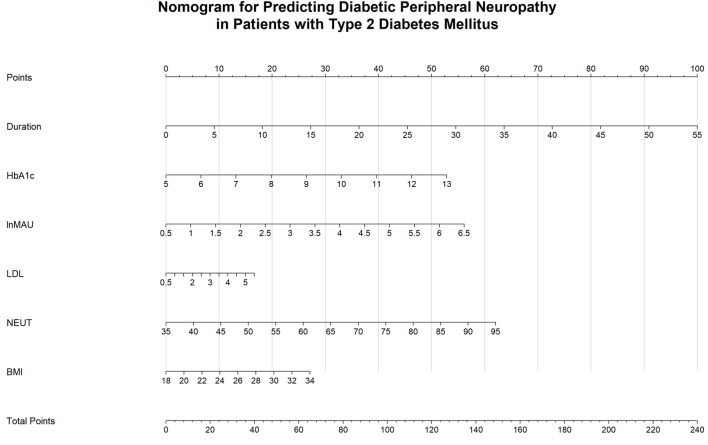
A DPN risk stratification nomogram constructed based on six independent predictive factors using multivariate logistic regression. DPN: diabetic peripheral neuropathy.

**Figure 6:**
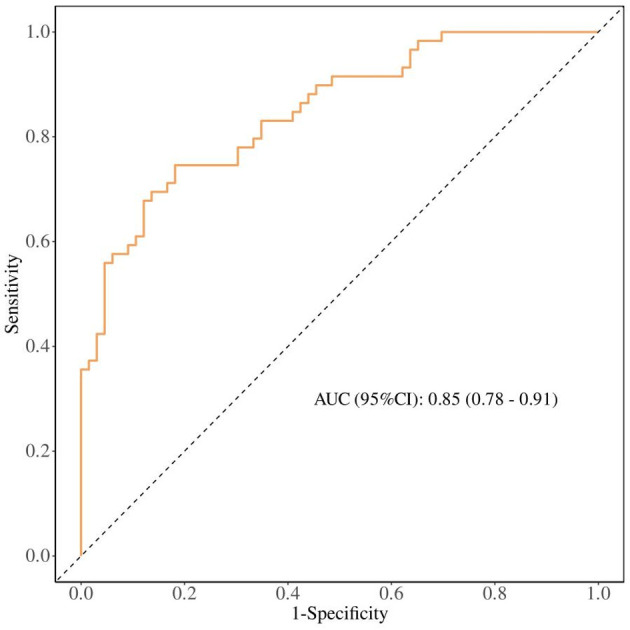
ROC curve analysis using a nomogram model. ROC: receiver operating characteristic.

Based on the six core independent predictive variables screened and verified by the optimal XGBoost model (duration of diabetes, HbA1c, ln-MAU, LDL-C, NEUT%, and BMI), a visualized DPN risk stratification nomogram was further constructed for convenient bedside clinical application (Fig. 5). Notably, the nomogram took the feature framework of the superior XGBoost model as the core basis, rather than conventional LR screening results, which ensures that the simplified clinical tool retains the optimal risk stratification logic of the ML model. The nomogram is applied clinically as follows: for each predictor variable, the corresponding scale value is identified and projected vertically onto the points axis to obtain an individual score; the sum of all individual scores yields a total score, which is then mapped onto the probability axis at the bottom of the nomogram to determine the corresponding predicted probability of DPN. All these line graph models demonstrated good discrimination, with an AUC of 0.85 on the validation set (95% CI: 0.78–0.91) (Fig. 6). As a practical clinical illustration, a T2DM patient with a diabetes duration of 12 years (55 points), HbA1c of 9.5% (62 points), MAU of 120 mg/24 h (ln = 4.79; 58 points), LDL-C of 3.2 mmol/L (42 points), NEUT% of 72% (38 points), and BMI of 27.5 kg/m^2^ (35 points) would accumulate a total score of 290 points, corresponding to a predicted DPN probability of approximately 82%, thereby indicating high-risk status and warranting further evaluation by NCV studies for confirmatory diagnosis.

**Figure 7:**
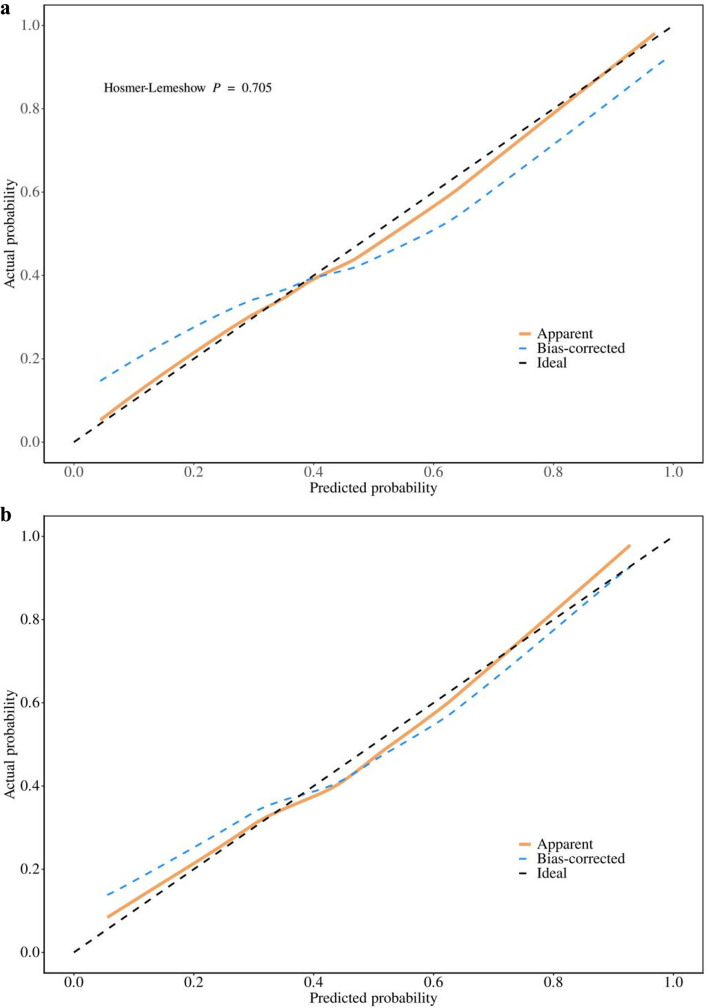
Line graph of the calibration curve. (a) Nomogram model. (b) XGBoost model.

**Figure 8:**
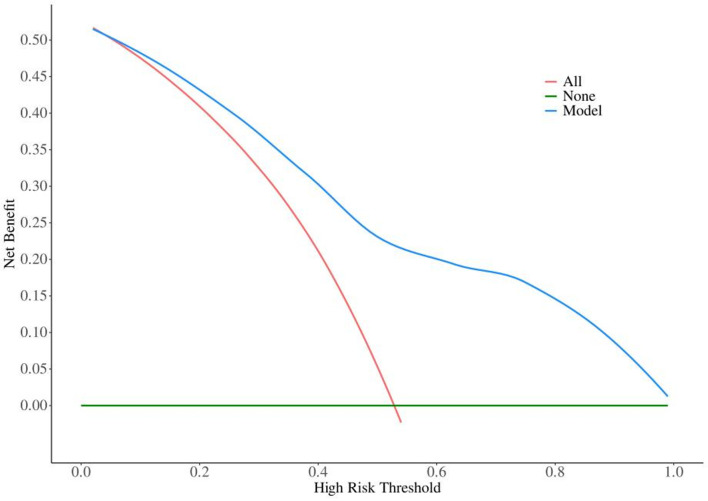
Decision curve analysis (DCA).

Calibration assessment demonstrated satisfactory performance of the nomogram in the validation set, with the calibration curve closely approximating the 45° ideal reference line, a Hosmer–Lemeshow test P-value of 0.512 (P > 0.05), and a mean absolute error of only 0.038 (Fig. 7a). For the optimal XGBoost model, comprehensive calibration evaluation in the internal hold-out validation set yielded satisfactory results: calibration intercept = −0.021, calibration slope = 0.976, Brier score = 0.064. The XGBoost calibration plot (Fig. 7b) showed a high consistency between model-estimated risk and actual DPN prevalence, indicating excellent calibration performance of the XGBoost model. DCA (Fig. 8) further substantiated the clinical utility of the nomogram, demonstrating that, across a wide range of threshold probabilities from 10% to 90%, the use of this predictive model consistently yielded greater net clinical benefit compared with both the “treat-all” and “treat-none” default strategies, thereby providing clinicians with a scientifically robust and practically applicable decision-support instrument.

## Discussion

This study integrated three complementary variable selection strategies, including univariable screening, LASSO regression, and RF-based feature importance ranking, to screen core predictors for DPN based on clinical data from 180 hospitalized patients with T2DM. Six core predictive variables were finally identified: duration of diabetes, HbA1c, MAU, LDL-C, NEUT%, and BMI. On this basis, a multi-model comparison framework covering five commonly used algorithms was constructed for preliminary exploration of DPN risk stratification. The current study preliminarily explored the predictive performance of different models, interpreted the nonlinear predictive patterns of key features via SHAP analysis, and developed a visualized nomogram for auxiliary risk evaluation. The following discussion objectively interprets the biological rationality of predictive variables, model performance characteristics, preliminary clinical translation potential, and inherent study limitations.

All six screened predictive variables are consistent with the recognized pathophysiological mechanism of DPN occurrence and progression. Diabetes duration ranked first in the SHAP-based feature importance analysis, which is in line with findings from large-scale prospective cohort studies. Long-term persistent metabolic disorders can induce progressive axonal degeneration and myelin sheath injury through the polyol pathway overactivation, PKC activation, and cumulative oxidative stress, thereby increasing DPN risk with prolonged disease course [10]. As a classic biomarker reflecting long-term glycemic exposure, HbA1c exhibited an obvious threshold effect on DPN prediction. DPN risk increased sharply when HbA1c exceeded 8%, which is consistent with the longitudinal results of the DCCT/EDIC study confirming that intensive glycemic control effectively reduces diabetic peripheral nerve injury risk [21]. Moreover, the synergistic interaction between HbA1c and diabetes duration observed in this study indicates that sustained poor glycemic control combined with prolonged disease course may produce superimposed neurotoxic damage. MAU is not only an early marker of diabetic nephropathy but also a typical manifestation of systemic microvascular endothelial dysfunction. The S-shaped dose–response relationship between ln(MAU) and DPN risk, with a marked risk increase when MAU exceeded 50 mg/24 h, verifies the existence of a biological threshold for microvascular injury. Endothelial dysfunction can increase the permeability of endoneurial capillaries and cause neuronal ischemia and hypoxia, which explains the close pathological correlation between MAU elevation and DPN occurrence [4, 22].

Beyond these conventional metabolic and microvascular biochemical markers, speckle-tracking echocardiography (STE)-derived myocardial deformation parameters may further broaden the scope of integrated risk assessment among patients with diabetes. Accumulating evidence demonstrates that left ventricular global longitudinal strain (LV-GLS) can capture early subclinical myocardial damage in diabetic individuals even when left ventricular ejection fraction remains within the normal reference range [23]. Reduced LV-GLS reflects cumulative damage driven by diabetic microangiopathy, chronic low-grade inflammation, myocardial fibrosis and long-standing metabolic disturbances, and it predicts subsequent adverse cardiovascular events long before overt structural heart disease emerges [24]. Such cardiac strain indicators carry complementary predictive value alongside HbA1c, MAU, and circulating inflammatory indices. Combined assessment of peripheral neuropathy biomarkers and STE myocardial strain will support multi-organ risk stratification and facilitate more individualized risk screening for T2DM patients. Future ML models may incorporate LV-GLS and other myocardial deformation indices to improve the comprehensiveness of multi-dimensional risk prediction.

NEUT% was significantly higher in the DPN group than in the non-DPN group, and served as an independent inflammatory predictor for DPN. Chronic low-grade inflammation is now considered a key pathogenic factor of DPN. Neutrophils and their secreted inflammatory mediators, including myeloperoxidase, neutrophil elastase, interleukin-1β (IL-1β), tumor necrosis factor-α, and interleukin-6, can directly damage Schwann cells and endoneurial microvessels and activate complement-mediated neuroinflammatory responses [6]. A previous NHANES-based study also confirmed the positive correlation between systemic inflammatory markers and DPN risk, supporting the predictive value of peripheral inflammatory indicators for DPN [25]. Elevated LDL-C contributes to DPN progression by exacerbating neural microvascular atherosclerosis and reducing nerve tissue perfusion. Oxidized LDL can trigger Schwann cell apoptosis and demyelination via the TLR4/NF-κB inflammatory pathway, while lipid-lowering statin therapy has been reported to have potential neuroprotective effects, further verifying the pathogenic role of LDL-C in DPN [26, 27]. BMI affects DPN risk through multiple metabolic and mechanical pathways. Obesity-induced insulin resistance aggravates glucotoxicity, abnormal adipokine secretion impairs dorsal root ganglion neuron function, and excess body weight causes persistent mechanical tension on peripheral nerve axons, collectively accelerating nerve function damage ([28].

Multi-model comparative analysis showed that the XGBoost model yielded the highest AUC value in the internal hold-out validation set, with statistically better discriminatory ability than LR, SVM, and DT. No significant AUC difference was observed between XGBoost and RF, while XGBoost showed slightly better performance in other comprehensive evaluation metrics. Existing ML studies on diabetic complications have confirmed that ensemble learning models have unique advantages in capturing complex nonlinear interactions among clinical variables, with better predictive efficacy than traditional linear models [29]. The XGBoost algorithm adopts gradient boosting iteration, L1/L2 regularization, and random sampling strategies, which can effectively balance model bias and variance and reduce overfitting risk. The minor AUC difference between the training set and validation set in this study preliminarily verified the stable internal performance of the model after Bayesian hyperparameter optimization. In contrast, SVM and single DT models are more susceptible to sample size and hyperparameter settings, showing limited generalization ability in small-sample cohorts. NRI and IDI analyses further demonstrated that XGBoost provided incremental discriminatory and reclassification value compared with conventional LR, offering more refined risk stratification information [30]. It is necessary to emphasize that the above performance differences are only preliminary results based on single-center internal validation with a limited sample size. The model performance ranking cannot be generalized, and relevant conclusions need further verification.

The poor interpretability of ML models is a key factor restricting their clinical transformation [16]. This study adopted SHAP analysis to quantify the independent predictive contribution and interaction effect of each feature, realizing transparent interpretation of model prediction results. Based on rigorous game theory algorithms, SHAP analysis can objectively evaluate feature importance and reveal nonlinear threshold effects that cannot be identified by traditional linear models [31]. The study found that HbA1c below 7% had a protective effect against DPN, which is highly consistent with the clinical glycemic control target for T2DM, confirming the clinical rationality of the model threshold. Individualized SHAP waterfall plots can quantitatively identify the dominant risk factors of DPN in a single patient, which helps clinicians formulate targeted individualized intervention measures and improve the accuracy of clinical decision-making [15, 32].

Considering that the XGBoost algorithm cannot be directly applied to rapid bedside clinical evaluation due to computational limitations, this study constructed a visualized DPN risk prediction nomogram based on the six core predictive variables screened by the optimal model. As a mature clinical prediction tool, nomograms can convert complex model algorithms into intuitive scoring systems, facilitating rapid risk assessment by clinicians without professional statistical background [33]. The nomogram showed acceptable discrimination, calibration, and clinical net benefit in internal validation, with the calibration curve fitting well with the ideal reference line and favorable Hosmer–Lemeshow test results [34]. DCA further confirmed that the nomogram could bring stable net clinical benefit within a wide threshold range, avoiding the clinical risk of missed diagnosis and over-screening [35]. In addition, all predictive indicators included in the nomogram are routine clinical examination items without additional detection cost, ensuring good clinical operability. Nevertheless, the nomogram is only an exploratory research tool based on internal validation, and its clinical application value needs further external verification.

Several limitations of the present study warrant careful consideration when interpreting the findings. First, the single-center, retrospective, cross-sectional design and the relatively modest sample size (n = 180) constrain the generalizability of the results; only internal hold-out validation was completed in this study, and external multicenter validation is urgently required before clinical application. Second, the study adopted mixed DPN diagnostic modalities: partial patients were diagnosed via gold-standard NCV testing, while mild and partial moderate DPN cases were diagnosed based solely on standardized clinical examination criteria. The inconsistent diagnostic methods may introduce population heterogeneity and limit the comparability of this study’s results with studies adopting unified NCV-based diagnostic criteria. Third, several emerging biomarkers with demonstrated potential for the early detection of DPN including homocysteine, intraepidermal nerve fiber density, and corneal nerve morphological parameters were not incorporated into the current predictive framework; future studies should consider integrating these indicators to further enhance model performance. Fourth, the optimal hyperparameters identified for the ML models may be sensitive to the composition of the training sample, and their stability should be re-evaluated when applying these models to cohorts with different demographic characteristics.

This retrospective cross-sectional study preliminarily constructed a DPN risk stratification framework combining traditional statistics and ML algorithms based on 180 T2DM inpatients. Six core independent predictive indicators for DPN were systematically screened and verified, including diabetes duration, HbA1c, MAU, LDL-C, NEUT%, and BMI. On the basis of multi-model comparison and internal validation, the XGBoost model showed relatively good preliminary predictive performance and stable internal generalization ability, with incremental clinical value compared with traditional LR. SHAP analysis clarified the nonlinear predictive rules and critical threshold effects of core indicators, resolving the interpretability defects of traditional ML models. The simplified visualized nomogram also achieved favorable internal predictive efficacy and clinical net benefit. Notably, all findings of this study are exploratory and preliminary. Restricted by the small sample size and single-center design, the model lacks external multicenter validation, and the current performance results and model ranking are not sufficiently stable. The prediction model and nomogram cannot be used for formal clinical diagnosis and bedside decision-making. Large-sample, multicenter external validation is essential in future studies to further verify the stability and generalizability of the model before clinical implementation. This study only provides a preliminary methodological reference for early risk identification and auxiliary management of DPN in T2DM patients.

## Data Availability

The data that support the findings of this study are available from the corresponding author upon reasonable request.

## Abbreviations

BMI: body mass index
DCA: decision curve analysis
DPN: diabetic peripheral neuropathy
DT: decision tree
FPG: fasting plasma glucose
HbA1c: glycated hemoglobin
HDL-C: high-density lipoprotein cholesterol
HGB: hemoglobin
LASSO: least absolute shrinkage and selection operator
LDL-C: low-density lipoprotein cholesterol
LR: logistic regression
MAU: microalbuminuria
NEUT%: neutrophil percentage
PLT: platelet count
RF: random forest
ROC: receiver operating characteristic
SHAP: SHapley Additive exPlanations
SVM: support vector machine
T2DM: type 2 diabetes mellitus
TC: total cholesterol
TG: triglycerides
XGBoost: extreme gradient boosting

## References

[1] Magliano DJ, Boyko EJ, IDF Diabetes Atlas 10th edition scientific committee IDF Diabetes Atlas.

[2] Wang L, Peng W, Zhao Z, Zhang M, Shi Z, Song Z, Zhang X (2021). Prevalence and treatment of diabetes in China, 2013-2018. JAMA.

[3] Pop-Busui R, Boulton AJ, Feldman EL, Bril V, Freeman R, Malik RA, Sosenko JM (2017). Diabetic neuropathy: a position statement by the American diabetes association. Diabetes Care.

[4] Vinik AI, Nevoret ML, Casellini C, Parson H (2013). Diabetic neuropathy. Endocrinol Metab Clin North Am.

[5] Armstrong DG, Boulton AJM, Bus SA (2017). Diabetic foot ulcers and their recurrence. N Engl J Med.

[6] Zakin E, Abrams R, Simpson DM (2019). Diabetic neuropathy. Semin Neurol.

[7] Brownlee M (2005). The pathobiology of diabetic complications: a unifying mechanism. Diabetes.

[8] Giacco F, Brownlee M (2010). Oxidative stress and diabetic complications. Circ Res.

[9] Fernyhough P, Roy Chowdhury SK, Schmidt RE (2010). Mitochondrial stress and the pathogenesis of diabetic neuropathy. Expert Rev Endocrinol Metab.

[10] Callaghan BC, Cheng HT, Stables CL, Smith AL, Feldman EL (2012). Diabetic neuropathy: clinical manifestations and current treatments. Lancet Neurol.

[11] Perkins BA, Olaleye D, Zinman B, Bril V (2001). Simple screening tests for peripheral neuropathy in the diabetes clinic. Diabetes Care.

[12] Bril V, Tomioka S, Buchanan RA, Perkins BA, m TSG (2009). Reliability and validity of the modified Toronto Clinical Neuropathy Score in diabetic sensorimotor polyneuropathy. Diabet Med.

[13] Christodoulou E, Ma J, Collins GS, Steyerberg EW, Verbakel JY, Van Calster B (2019). A systematic review shows no performance benefit of machine learning over logistic regression for clinical risk stratification models. Journal of clinical epidemiology.

[14] Martin CL, Albers JW, Pop-Busui R, DCCT/EDIC Research Group (2014). Neuropathy and related findings in the diabetes control and complications trial/epidemiology of diabetes interventions and complications study. Diabetes Care.

[15] Rajpurkar P, Chen E, Banerjee O, Topol EJ (2022). AI in health and medicine. Nat Med.

[16] Obermeyer Z, Emanuel EJ (2016). Predicting the future - big data, machine learning, and clinical medicine. N Engl J Med.

[17] Eley A, Hlaing TT, Breininger D, Helforoush Z, Kachouie NN (2025). Monte Carlo gradient boosted trees for cancer staging: a machine learning approach. Cancers (Basel).

[18] Chen L, Shao X, Yu P (2024). Machine learning risk stratification models for diabetic kidney disease: systematic review and meta-analysis. Endocrine.

[19] Shi G, Gao Z, Zhang Z, Jin Q, Li S, Liu J (2025). Machine learning-based risk stratification model for neuropathic foot ulcers in patients with diabetic peripheral neuropathy. Journal of diabetes investigation.

[20] Nohara Y, Matsumoto K, Soejima H, Nakashima N (2022). Explanation of machine learning models using shapley additive explanation and application for real data in hospital. Comput Methods Programs Biomed.

[21] Diabetes C, Complications Trial Research G, Nathan DM, Genuth S, Lachin J, Cleary P, Crofford O (1993). The effect of intensive treatment of diabetes on the development and progression of long-term complications in insulin-dependent diabetes mellitus. N Engl J Med.

[22] Ziegler D, Papanas N, Vinik AI, Shaw JE (2014). Epidemiology of polyneuropathy in diabetes and prediabetes. Handb Clin Neurol.

[23] Holland DJ, Marwick TH, Haluska BA, Leano R, Hordern MD, Hare JL, Fang ZY (2015). Subclinical LV dysfunction and 10-year outcomes in type 2 diabetes mellitus. Heart.

[24] Sonaglioni A, Barlocci E, Adda G, Esposito V, Ferrulli A, Nicolosi GL, Bianchi S (2022). The impact of short-term hyperglycemia and obesity on biventricular and biatrial myocardial function assessed by speckle tracking echocardiography in a population of women with gestational diabetes mellitus. Nutr Metab Cardiovasc Dis.

[25] Upadhyayula SK, Ubaru S, Raajeshwi P, Ajavindu CN, Rao AB (2024). Neutrophil-lymphocyte ratio and urine albumin-creatinine ratio as indicators of microvascular complications in type 2 diabetes mellitus: a cross-sectional study. Cureus.

[26] Ishibashi F, Taniguchi M, Kosaka A, Uetake H, Tavakoli M (2019). Improvement in neuropathy outcomes with normalizing HbA(1c) in patients with type 2 diabetes. Diabetes Care.

[27] Knopp RH, d'Emden M, Smilde JG, Pocock SJ (2006). Efficacy and safety of atorvastatin in the prevention of cardiovascular end points in subjects with type 2 diabetes: the Atorvastatin Study for Prevention of Coronary Heart Disease Endpoints in non-insulin-dependent diabetes mellitus (ASPEN). Diabetes Care.

[28] Kazamel M, Stino AM, Smith AG (2021). Metabolic syndrome and peripheral neuropathy. Muscle Nerve.

[29] Contreras I, Vehi J (2018). Artificial intelligence for diabetes management and decision support: literature review. J Med Internet Res.

[30] Pencina MJ, D'Agostino RB, D'Agostino RB, Vasan RS (2008). Evaluating the added predictive ability of a new marker: from area under the ROC curve to reclassification and beyond. Stat Med.

[31] Rudin C (2019). Stop explaining black box machine learning models for high stakes decisions and use interpretable models instead. Nat Mach Intell.

[32] Holzinger A, Langs G, Denk H, Zatloukal K, Muller H (2019). Causability and explainability of artificial intelligence in medicine. Wiley Interdiscip Rev Data Min Knowl Discov.

[33] Balachandran VP, Gonen M, Smith JJ, DeMatteo RP (2015). Nomograms in oncology: more than meets the eye. Lancet Oncol.

[34] Steyerberg EW, Vickers AJ, Cook NR, Gerds T, Gonen M, Obuchowski N (2010). Assessing the performance of risk stratification models: a framework for traditional and novel measures. Epidemiology (Cambridge, Mass).

[35] Vickers AJ, Van Calster B, Steyerberg EW (2016). Net benefit approaches to the evaluation of prediction models, molecular markers, and diagnostic tests. BMJ.

